# Hereditary Aspects of Colorectal Cancer: Mismatch Repair Genes Drive Lynch Syndrome

**Published:** 2018-04-01

**Authors:** Heather Hampel, Michael J. Hall

**Affiliations:** 1 The Ohio State University, Columbus, Ohio;; 2 Fox Chase Cancer Center, Philadelphia, Pennsylvania

## Abstract

Lynch syndrome is the most common inherited form of colorectal cancer, affecting approximately 1 in 279 individuals, or 1.2 million people in the United States. It’s also associated with a predisposition to endometrial, ovarian, and stomach cancers, among others. Advanced practitioners should be familiar with the genetics for this syndrome, as well as guidelines for screening and managing this common hereditary predisposition.

The prevalence of Lynch syndrome (previously known as hereditary nonpolyposis colorectal cancer [HNPCC]), the most common inherited form of colorectal cancer (CRC), is approximately 1 in 279 individuals, or 1.2 million people in the United States. Lynch syndrome, which is characterized by microsatellite instability (MSI), is also associated with a predisposition to endometrial, ovarian, and stomach cancers, among others. Advanced practitioners should be familiar with this condition, the associated genetics, and recommendations for testing and intervention, according to Heather Hampel, MS, LGC, of The Ohio State University, Columbus, and Michael J. Hall, MD, MS, of Fox Chase Cancer Center, Philadelphia, who discussed this complex disorder at JADPRO Live 2017.

"The important thing about Lynch syndrome is that most of the cancers are preventable," said Ms. Hampel. "So, as long as we start colonoscopies early enough and do them frequently enough, we believe we can keep these patients from getting colon cancer."

## KEY GENES

Lynch syndrome is caused by a mutation in one of four mismatch repair genes—*MLH1*, *MSH2* (including terminal EPCAM deletions), *MSH6*, and *PMS2*, with a mutation in *MLH1* or *MSH2* conferring much higher cancer risks. The syndrome is autosomal dominant, which means that individuals who have a parent with Lynch syndrome have a 50% chance that they will inherit the gene mutation and have Lynch syndrome too. Therefore, in individuals with Lynch syndrome, every cell in the body has one nonworking copy of the gene. A working copy of the gene from the other parent prevents the development of cancers for many years. The odds are high, however, that over the years, when an at-risk cell in the colon or the endometrium is dividing, "they are just going to acquire a mistake in the working copy; a so-called second hit, and now we have a cell with no more working mismatch repair gene," said Ms. Hampel. "Once both copies of the repair gene are not working, the cell will begin to accumulate genetic mutations. When any one cell gets enough mistakes or mutations, that cell will become a cancer."

Because of the lower cancer risks associated with mutation of *MSH6* or *PMS2*, the family history may not be as striking, and therefore family history alone is not adequately sensitive to identify patients with Lynch syndrome.

The revised Bethesda guidelines (Umar et al., 2004) have largely replaced the Amsterdam II criteria (Vasen, Watson, Mecklin, & Lynch, 1999), which relied heavily on family history, for the identification of individuals with Lynch syndrome who should undergo tumor testing for MSI. According to the revised Bethesda guidelines, the following features should prompt tumor testing and referral to a genetic counselor:

- A diagnosis of CRC before age 50
- Synchronous or metachronous CRC, or other Lynch syndrome–associated tumors regardless of age
- CRC with a diagnosis of MSI-high histology before age 60
- CRC with ≥ one first-degree relative with a Lynch syndrome–associated tumor, with one cancer diagnosis before age 50
- CRC with ≥ two first-degree or second-degree relatives with a Lynch syndrome–associated tumor, regardless of age

The PREMM_5_ Lynch syndrome prediction model predicts the likelihood of a germline mutation in any of the four genes associated with Lynch syndrome (PREMM, 2017). The original model recommended referral to a genetic counselor if the chance was 5% or greater. A newer proposal lowers the threshold to 2.5% (Kastrinos et al., 2017).

## SCREENING FOR LYNCH SYNDROME

Tumor tests to screen for Lynch syndrome include testing for the presence of MSI, immunohistochemistry (IHC) staining, and methylation/*BRAF* V600E testing. Microsatellite instability testing is performed on DNA extracted from the tumor and normal tissue, and requires a molecular laboratory for this purpose. Microsatellite instability testing is positive in about 15% of CRC cases and in 77% to 89% of Lynch syndrome cases, "so it’s not diagnostic, but it does select a group of patients that is more likely to have Lynch syndrome and could benefit from germline genetic testing," Ms. Hampel indicated.

Far more commonly adopted at hospitals is IHC testing, in which tumor samples are stained with the antibodies to the four mismatch repair proteins. "A normal result is the presence of all four proteins (low probability of MSI), which occurs about 80% of the time," according to Ms. Hampel. If IHC staining is absent for *MLH1* and *PMS2*, the most common cause is acquired methylation of the *MLH1* protein. Testing for a *BRAF* V600E mutation is a surrogate for *MLH1* methylation, as *BRAF* mutation correlates with methylation 69% of the time. Tumors that show neither *BRAF* V600E mutation nor MLH1 promoter methylation are most often caused by an inherited mutation, and therefore require referral to a genetic counselor.

The second most common result of IHC staining is absence of *MSH2* or *MSH6*, in approximately 2%, and is strongly indicative of Lynch syndrome due to an *MSH2* or *MSH6* gene mutation. Less commonly, but also strongly suggestive of Lynch syndrome is the absence of *MSH6* or *PMS2* alone in a tumor. In these cases, referral to a genetic counselor is always warranted.

Routine tumor testing criteria includes performing the MSI or IHC test on either (1) all patients with CRC or (2) those with CRC diagnosed before age 70 and those with CRC diagnosed at age 70 and older who meet the revised Bethesda guidelines. Tumor screening for endometrial cancer patients using MSI or IHC can be performed on (1) all patients with endometrial cancer; (2) those diagnosed with endometrial cancer before age 60; or (3) those patients with endometrial cancer who meet the modified Bethesda guidelines.

## POLYPOSIS SYNDROMES

**Figure 1:**
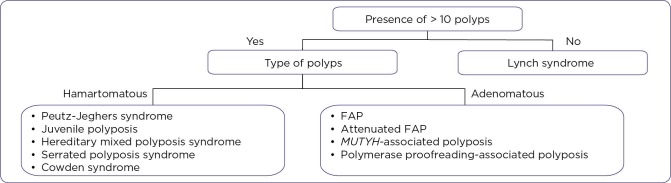
Flowchart for hereditary colon cancer: differential diagnosis. FAP = familial adenomatous polyposis.

The presence of more than 100 adenomas throughout the colon is characteristic of familial adenomatous polyposis (FAP) syndrome (Figure 1). Even with the obvious diagnosis, genetic testing is still useful in such patients. "If we can do genetic testing and find the mutation, we can test the kids so they know whether they need to start their colonoscopies early or not, and when I say ’early’ we are talking potentially 10- and 11-year-olds," she said. "Some families even elect to test newborns for FAP if they would like to have their infant screened for hepatoblastoma (a rare tumor that is slightly more common in individuals with Lynch syndrome) from birth to age 5."

With a weaker form of FAP—attenuated FAP—polyp counts tend to be 20 to 100. "In the low counts it gets a little confusing about whether to refer or not," she said. "We generally refer anyone with more than 10 adenomas to our clinic, and lots of them test negative when you test them for these polyp genes, but every once in a while one of them tests positive."

*MUTYH*-associated polyposis is characterized by 20 to hundreds of adenomatous polyps, and is often detected through the use of colon cancer gene panels. Out of 50 Caucasians from Western Europe, 1 will be a carrier of a *MUTYH* mutation. Polyposis occurs only in individuals who have inherited two *MUTYH* mutations, one from each parent.

Polymerase proofreading-associated polyposis is a newer syndrome that is still being defined. It is caused by mutations in the *POLD1* and *POLE* genes "and these make ultra-hypermutated tumors if tumor sequencing is done and…they seem to make dozens of adenomas," Ms. Hampel indicated.

Hamartomatous polyposis syndromes consist of Peutz-Jeghers syndrome (*STK11 gene*), juvenile polyposis syndrome (*BMPR1A* and *SMAD4* genes), and serrated polyposis syndrome (no major genes known). These syndromes represent fewer than 1% of all colon cancers.

Mixed polyposis syndromes include hereditary mixed polyposis syndrome (*GREM1* gene), seen mostly in individuals of Ashkenazi Jewish ancestry, and Cowden syndrome (*PTEN* gene), an inherited syndrome characterized by multiple noncancerous growths and increased risk of breast, endometrial, colorectal, and thyroid cancers.

## GENETIC TESTING

*Lynch syndrome*. Clinical criteria for testing for Lynch syndrome should be offered to patients who meet the revised Bethesda or Amsterdam II criteria, patients with a colorectal or endometrial cancer diagnosis before age 50 or with abnormal MSI or IHC testing at any age, individuals with a PREMM_5_ score > 2.5%, and individuals who have a known family history of Lynch syndrome.

*Polyposis*. Testing for the adenomatous polyposis syndromes is recommended in patients who have more than 10 adenomas. Testing for hamartomatous polyposis syndromes should be performed in patients with two Peutz-Jeghers polyps, five juvenile polyps, or a patient of Ashkenazi Jewish ancestry with multiple mixed polyps.

## WHICH TESTS?

The cost of tumor screening tests (MSI, IHC) has dropped to $500 or less. Next-generation gene testing panels that include as many as 80 genes are now available for $250 to $4,000, with little out-of-pocket cost to the patient. "Due to the overlap between a lot of the polyposis syndromes and Lynch syndrome, panels really are the preferred approach for colon cancer genetic testing," said Ms. Hampel. All patients with early-onset CRC should be tested with a broad cancer gene panel because a recent study found that 16% of these patients have a mutation in cancer susceptibility genes and some were in genes that have not previously been associated with cancer (Pearlman et al., 2017).

## MANAGING LYNCH SYNDROME

Starting colonoscopy at age 20 to 25, and repeating examination every 1 to 2 years, is essential to the management of Lynch syndrome, said Dr. Hall (Table 1). Although intensive screening reduces the risk of CRC in affected individuals, the risk is not eliminated entirely. "For those patients who may not accept that risk, or may have other reasons, we do still think of colectomy as a possible option for these patients," he said. "Also, in this syndrome you see polyps that are frankly just not amenable to good surveillance with colonoscopy."

**Table 1:**
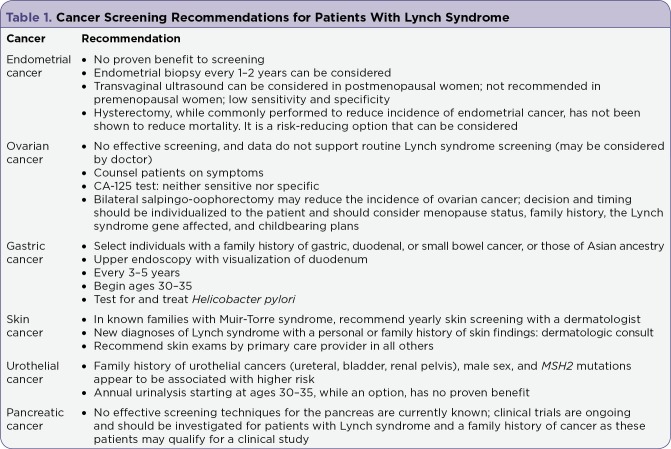
Cancer Screening Recommendations for Patients With Lynch Syndrome

An underrecognized chemopreventive agent in patients with Lynch syndrome is aspirin. In the CAPP2 study, patients with Lynch syndrome who were randomized to aspirin at 600 mg/day, and were adherent for at least 2 years, had a 63% reduction in the rate of incident CRC and a 51% reduction in the rate of non-CRC Lynch syndrome cancers after 4 or more years of follow-up (Burn et al., 2011). Expert groups are awaiting confirmatory studies before endorsing aspirin for this indication, and toxicities at this dosage of aspirin are a concern, said Dr. Hall.

Guidelines for the management of other Lynch syndrome risks are evolving, as more is learned about the syndrome and individual genes. Guidelines should be integrated with personal and family history and the clinician’s best clinical judgment. For women, oophorectomy can reduce the incidence of ovarian cancer. A total abdominal hysterectomy is a risk-reducing option to lower the incidence of endometrial cancer, but has no mortality benefit. Most recent estimates have described the risks of endometrial cancer and ovarian cancer to be highest with mutations in *MSH2*.

*MLH1* and *MSH2* are the highest-risk genes for gastric cancer, the incidence of which has plummeted in the United States owing to better storage and preparation of food and a reduced incidence of *Helicobacter pylori* infection. Upper endoscopy with visualization of the duodenum is recommended beginning at ages 30 to 35 and at an interval of every 3 to 5 years to screen for gastric cancer (Vasen et al., 2013) for individuals with Lynch syndrome who have a family history of stomach cancer or are of Asian descent. Testing for and treating *H. pylori* is also recommended.

Yearly skin screening by a dermatologist is recommended with a family history of Muir-Torre syndrome, a variant of Lynch syndrome (South et al., 2008). Otherwise, a dermatologic consult is called for in patients with a new diagnosis of Lynch syndrome and a personal or family history of skin findings.

## IMMUNOTHERAPIES IN COLORECTAL CANCER

Lynch syndrome is now informing the treatment of CRC, Dr. Hall said. The mismatch repair pathway is activated in some individuals with CRC who do not have Lynch syndrome. The two broad categories of deficient mismatch repair in colorectal tumors are germline plus somatic mismatch repair gene mutations (as in Lynch syndrome) and somatic plus somatic mismatch repair gene mutations. These are targetable with inhibitors of programmed cell death protein 1 (PD-1) and its ligand (PD-L1).

Mismatch repair–deficient tumors are thought to be responsive to immunotherapies because they have a high level of tumor-infiltrating lymphocytes and a higher mutational burden, which predict for immunotherapy response, Dr. Hall explained. Although the objective response rate to immunotherapy is high in mismatch repair–deficient tumors, few patients experience complete response. With most patients having partial response or stable disease as a best response, the optimal duration of therapy is not yet known.

Several biomarkers are predictive and prognostic in the treatment of CRC. The first marker discovered that drove clinical decisions in CRC was *KRAS*. Patients with RAS wild type were observed to have better responses to EGFR-directed therapy than those with *KRAS* mutations, who had no response or experienced tumor progression on such therapy; this led to a recommendation to avoid EGFR inhibitors in patients with *KRAS* mutations, a recommendation that was expanded to include *NRAS* and *BRAF* mutations. The absence of mutations in *KRAS*, *NRAS*, and *BRAF* predicts response to EGFR inhibitors (cetuximab [Erbitux] and panitumumab [Vectibix]), although the correlation is not 100%.

"*KRAS* mutations on the left side [of the colon] seem to respond much more robustly to anti-EGFR therapy than those on the right side," said Dr. Hall, "and this was shown both through some retrospective data from CALGB 80403 (Bokemeyer et al., 2015), where survival was getting close to double, and also in a large multitrial retrospective database called ACCENT" (Arnold et al., 2017).
